# Mortality and Causes of Death After Metabolic Bariatric Surgery in Older Patients

**DOI:** 10.1007/s11695-026-08487-7

**Published:** 2026-01-12

**Authors:** Peter Gerber, Giola Santoni, My von Euler-Chelpin, Joonas H. Kauppila, Dag Holmberg

**Affiliations:** 1 Saint Göran Hospital, Stockholm, Sweden; 2 Karolinska Institutet, Stockholm, Sweden; 3 University of Copenhagen, Copenhagen, Denmark; 4 Oulu University Hospital, Oulu, Finland

**Keywords:** Gastric bypass, Sleeve gastrectomy, Obesity, Roux-en-y, Weight loss

## Abstract

**Background:**

Metabolic bariatric surgery leads to improved life expectancy in younger individuals, but whether older (> 60 years) individuals benefit from metabolic bariatric surgery is uncertain. This study examined mortality and causes of death in patients with metabolic bariatric surgery at age 60 years or older.

**Materials and Methods:**

This was a population-based matched cohort study based on all healthcare in Denmark, Finland, and Sweden between 1996 and 2024. All patients who had primary metabolic bariatric surgery at age > 60 years were included and exactly matched 1:5 to comparison individuals of the same age, sex, country, and calendar year with non-operative treatment for obesity. Cox regression provided hazard ratios with 95% confidence intervals for mortality adjusted for multiple obesity-related diseases and frailty.

**Results:**

In total, 3879 (16.7%) patients with metabolic bariatric surgery and 19395 (83.3%) patients with non-operative treatment for obesity were included and followed for 176632 person-years. The cumulative mortality was 17.5% (*n* = 677) among operated patients compared to 23.5% (*n* = 4536) in the non-operated. In adjusted analyses, metabolic bariatric surgery was associated with 32% decreased mortality (HR 0.68, 95% CI 0.63–0.73). The results were consistent in patients of age > 60–70 years at the time of surgery, but there was no apparent benefit in patients operated at age > 70 years (HR 1.14, 95% CI 0.89–1.47). Operated patients were less likely to die from cardiovascular disease (57.6% versus 65.8%, *p* < 0.001), but other causes of death were similarly distributed between the groups.

**Conclusion:**

Metabolic bariatric surgery may decrease mortality in older patients with severe obesity.

**Supplementary Information:**

The online version contains supplementary material available at 10.1007/s11695-026-08487-7.

## Introduction

Obesity is a global health problem that currently affects 30–40% of adults in high-income countries [1]. Obesity is associated with several diseases, mainly diabetes, hypertension, cardiovascular diseases, cancer, and psychiatric disorders, as well as reduced overall survival [2, 3]. Individuals with severe obesity (body mass index ≥ 35 kg/m^2^) are eligible for metabolic bariatric surgery, which induces rapid, pronounced and long-lasting weight loss, and in many cases resolution of obesity-related diseases [4–11]. Multiple studies have demonstrated the association between metabolic bariatric surgery and prolonged life expectancy, but have generally been conducted in younger patients [4, 7, 8, 12].

There is controversy whether metabolic bariatric surgery should be recommended to older individuals (> 60 years) with severe obesity, as the complication rate may be increased and the weight loss decreased compared to bariatric surgery performed in the young [13, 14]. Few studies have examined the potential long-term benefits of metabolic bariatric surgery in individuals age > 60 years. Using nationwide data from three Nordic countries, we set out to assess mortality and causes of death in patients who had bariatric surgery at age 60 years or older in comparison to patients with non-operative care for obesity.

## Materials and Methods

### Design

This was a population-based matched cohort study including data from three entire Nordic countries. The study period varied between the participating countries: Denmark, July 1, 1996-December 31, 2018; Finland, January 1, 1989-December 31, 2018; Sweden, January 1, 1989-December 31, 2024. Start of follow-up occurred with the introduction of ICD-9 in Finland and Sweden and with the introduction of operation codes for metabolic bariatric surgery in Denmark. All data used in the study were retrieved from nationwide healthcare databases, i.e., the patient registries, cancer registries, and cause of death registries of the three countries. Approvals were obtained from the relevant authorities in each country.

### Exposure

The study population was retrieved from the patient registries, which prospectively collect data on all in-hospital and specialized out-patient healthcare (not primary care), including individual level data on diagnoses and procedures. All patients that had undergone primary metabolic bariatric surgery, i.e., gastric bypass, sleeve gastrectomy, gastric banding, vertical banded gastroplasty, duodenal switch with biliopancreatic diversion, or other metabolic bariatric procedures (Supplementary Table 1), at age > 60 years in any of the three countries were considered exposed. These patients were compared to patients diagnosed with obesity (Supplementary Table 2) by a physician and not treated with metabolic bariatric surgery, i.e., non-operative treatment. After exact matching by country, age, sex, and calendar year, up to 5 patients with non-operative treatment for obesity were randomly selected for each operated patient. If 10 or less matching patients with non-operative treatment for obesity were available for a specific operated patient, matching age was extended up to a maximum of ± 2 years of age. Patients with a history of neoplasia were excluded.

The patient registries of the Nordic countries have been extensively validated overall with positive predictive values of diagnoses ranging from 73%−88% in Denmark [15], 75%−99% in Finland [16], and 85%−95% in Sweden [17]. Metabolic bariatric surgery has been specifically validated in Sweden, where a study of 938 patients showed 97% concordance between reviewed operation charts and data from the patient registry [18].

### Endpoints

The outcome was death due to any cause, which was identified in the cause of death registries. Additionally, we described the main cause of death for operated and non-operated patients. The cause of death registries contain virtually complete and continuously updated information on date of death and cause(s) of death in all three countries based on an assessment by the physician confirming the death [19, 20].

### Confounders

We identified several relevant confounders potentially associated with both a propensity of undergoing/not undergoing metabolic bariatric surgery and mortality: diabetes (yes or no), hypertension (yes or no), peripheral vascular disease (yes or no), chronic obstructive pulmonary disease (yes or no), renal disease (yes or no), and cardiovascular disease (yes or no). Additionally, we included frailty-related diagnoses: deep vein thrombosis or pulmonary embolism (yes or no), pneumonia (yes or no), and number of previous hospital admissions (continuous). All confounders were identified through a two-year historical search in the patient registries (Supplementary Table 3).

### Statistical Analysis

Operated patients entered the study on the date of primary metabolic bariatric surgery, with their corresponding matched comparison patients entering on the same date. Patients were followed until date of death or the end of the study period in each participating country, whichever occurred first. Multivariable Cox regression analysis was used to calculate hazard ratios (HR) with 95% confidence intervals (CI) for all-cause mortality. Standard errors were estimated using the clustered sandwich estimator. Two statistical models were used: one crude and one adjusted for all potential confounders with categorizations as described above (“Confounders” paragraph). The proportionality of the hazard was assessed by calculating the Schoenfeld residuals; the assumption of proportionality was not met, why the main analysis was presented stratified by follow-up period. Analyses were also stratified according to age (categorizations), sex (male or female), calendar year (divided by the median year), and diabetes (yes or no). Using Laplace regression, we calculated the difference in the 5th and 10th percentile of time to death with 95% CI in operated and non-operated patients adjusted by the confounders listed above [21]. In a post-hoc sub analysis, we excluded patients with a history of cardiovascular disease at baseline. Data management and analyses were conducted by a senior biostatistician (GS) in accordance with a detailed and pre-planned study protocol. The study followed the STROBE reporting guideline for cohort studies.

## Results

### Patients

We included 3879 patients with metabolic bariatric surgery at age > 60 years and 19,395 matched patients with non-operative treatment for obesity. Median age was 63 years (interquartile range, 62–65 years) and 64.1% were female in both groups (Table 1). Most operated patients underwent either gastric bypass (74.0%, *n* = 2871) or sleeve gastrectomy (15.4%, *n* = 599). Operated patients were more frequently diagnosed with diabetes and hypertension, but to a less degree cardiovascular disease. Variables associated with frailty were similarly distributed in both groups. 90-day mortality was 0.6% (*n* = 24) in operated patients and 0.4% (*n* = 74) in patients with non-operative treatment for obesity (*p* = 0.03). Causes of death within 90-days are reported in Supplementary Table 4.

**Table 1:** Characteristics among older patients with metabolic bariatric surgery and matched controls with nonoperative treatment for obesity

	Patients, No. (%)	
Characteristic	Nonoperative treatment	Metabolic bariatric surgery
Total	19395 (83.3)	3879 (16.7)
Sex		
Female	12430 (64.1)	2486 (64.1)
Male	6965 (35.9)	1393 (35.9)
Age, median (IQR), y	63 (62–65)	63 (62–65)
60–65	15,338 (79.1)	3072 (79.2)
66–70	3216 (16.6)	642 (16.6)
> 70	841 (4.3)	165 (4.3)
Year of entry, median (IQR)	2013 (2010–2017)	2013 (2010–2017)
Country		
Denmark	2190 (11.3)	438 (11.3)
Finland	4060 (20.9)	812 (20.9)
Sweden	13145 (67.8)	2629 (67.8)
Diabetes	4496 (23.2)	1454 (37.5)
Hypertension	6116 (31.0)	2147 (55.3)
Peripheral vascular disease	464 (2.4)	64 (1.6)
Chronic obstructive pulmonary disease	972 (5.0)	157 (4.0)
Renal disease	596 (3.1)	86 (2.2)
Cardiovascular disease	2719 (14.0)	381 (9.8)
Frailty		
Deep vein thrombosis or pulmonary embolism	224 (1.2)	32 (0.8)
Pneumonia	573 (3.0)	84 (2.2)
Previous hospital admissions*, median (IQR), no	3 (1–7)	4 (2–9)
90-day mortality	74 (0.4)	24 (0.6)
All-cause mortality	4536 (23.4)	677 (17.5)
Follow-up time, median (IQR), y	7.1 (3.3–10.9)	7.8 (4.0–11.3)

*Within two years of cohort entry

### Mortality

**Fig. 1:**
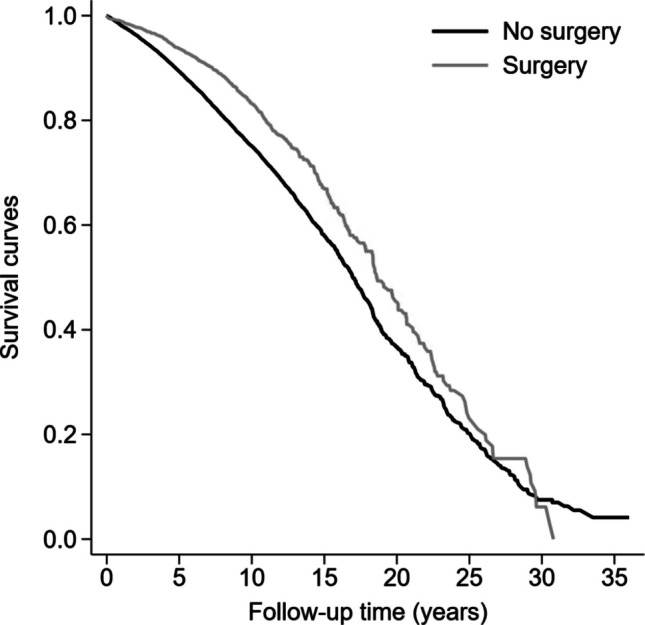
Cumulative survival as a function of time among older patients with metabolic bariatric surgery compared to nonoperative treatment for obesity

Patients were followed for median 7.7 years of follow-up, during which 5 213 (22.4%) died. The cumulative mortality was 17.5% (*n* = 677) in the operated patients compared to 23.4% (*n* = 4536) in the non-operated (Fig. 1). After adjustments, patients with metabolic bariatric surgery had a 32% decreased risk of mortality compared to matched patients (HR 0.68, 95% CI 0.63–0.73) (Table 2). Stratification by follow-up period revealed that the decreased risk associated with metabolic bariatric surgery was due to decreased mortality in the first 15 years after surgery (Table 2). With longer follow-up time, the adjusted hazard of death was similar in operated and non-operated patients. The association between surgery and mortality was apparent in patients operated at age > 60–64 (HR 0.64, 95% CI 0.58–0.71) and 65–70 years (HR 0.66, 95% CI 0.55–0.78), but there was no apparent benefit of surgery at age > 70 years (HR 1.23, 95% CI 0.90–1.67). Further stratified analyses by sex, country, calendar year, and diabetes largely verified a protective association between metabolic bariatric surgery and mortality (Table 2). The protective association was more pronounced in patients with diabetes (HR 0.56, 95% CI 0.48–0.66) compared to those without diabetes (HR 0.81, 95% CI 0.72–0.91) (Table 2). In the adjusted analysis of time to death, metabolic bariatric surgery was associated with 1.3 (95% CI 0.6–1.9) and 1.9 (95% CI 1.4–2.4) years longer life among the first 5% and 10% of people who died, respectively. A sub analysis restricted to patients with gastric bypass (*n* = 2871) with matched controls (*n* = 14355) showed 36% decreased mortality in operated patients (HR 0.64, 95% CI 0.58–0.71) (Supplementary Table 5). Similarly, the sub analysis in patients without cardiovascular disease at baseline (*n* = 17488), metabolic bariatric surgery remained associated with a decreased risk of all-cause mortality (HR 0.72, 95% CI 0.64–0.79).

**Table 2:** Mortality in older patients with metabolic bariatric surgery and non-operative treatment for obesity

	Non-operative treatment		Metabolic bariatric surgery			
					Hazard ratio (95% CI)	
Characteristic	Person-years	Deaths, No	Person-years	Deaths, No	Unadjusted	Adjusted*
Total	145745	4536	30887	677	0.69 (0.64–0.74)	0.68 (0.63–0.73)
Follow-up (years)						
< 1	18633	354	3746	42	0.59 (0.43–0.81)	0.62 (0.45–0.84)
1–5	72340	1757	15078	206	0.56 (0.49–0.65)	0.57 (0.49–0.66)
6–10	38916	1418	8566	230	0.74 (0.64–0.84)	0.71 (0.62–0.82)
11–15	12896	681	2900	123	0.80 (0.66–0.97)	0.75 (0.62–0.91)
> 15	2960	326	710	76	0.96 (0.75–1.23)	0.95 (0.74–1.20)
Sex						
Female	96182	2539	20438	367	0.66 (0.60–0.74)	0.65 (0.59–0.72)
Male	49563	1997	10560	310	0.72 (0.64–0.80)	0.72 (0.64–0.81)
Age						
≤ 65	118736	3221	25326	467	0.67 (0.61–0.73)	0.64 (0.58–0.71)
65–70	22086	914	4791	134	0.66 (0.55–0.78)	0.66 (0.55–0.78)
> 70	4923	401	881	76	1.08 (0.86–1.36)	1.14 (0.89–1.47)
Country						
Denmark	13879	361	2862	50	0.67 (0.51–0.88)	0.68 (0.51–0.90)
Finland	20464	643	4219	83	0.64 (0.52–0.77)	0.63 (0.51–0.77)
Sweden	111402	3532	23918	544	0.70 (0.64–0.76)	0.69 (0.63–0.75)
Calendar year						
≤ 2013	102427	3553	21925	576	0.74 (0.68–0.81)	0.72 (0.66–0.78)
> 2013	43318	983	9073	101	0.49 (0.40–0.59)	0.50 (0.41–0.62)
Diabetes						
No	114268	2941	19414	399	0.77 (0.70–0.85)	0.78 (0.71–0.86)
Yes	31477	1595	11584	278	0.46 (0.41–0.52)	0.56 (0.49–0.63)

*Adjusted for diabetes, hypertension, peripheral vascular disease, chronic obstructive pulmonary disease, renal disease, cardiovascular diseases, deep vein thrombosis or pulmonary embolism, pneumonia, number of previous hospital admissions

### Causes of Death

Cardiovascular disease (64.7%, *n* = 3375) was the most commonly occurring cause of death in both operated and non-operated, followed by cancer (26.0%, *n* = 1 354) and abdominal diseases (11.2%, *n* = 586) (Table 3). Operated patients were less likely to die from cardiovascular disease than the non-operated (57.6% versus 65.8%, *p* < 0.001)), but otherwise the causes of death were overall similarly distributed.

**Table 3:** Causes of death in older patients with metabolic bariatric surgery and non-operative treatment for obesity

	Deaths, No. (%)	
Cause of death	Non-operative treatment	Metabolic bariatric surgery
Total	4536	677
Cardiovascular	2985 (65.8)	390 (57.6)
Cancer	1161 (25.6)	193 (28.5)
Infections	447 (9.9)	61 (9.0)
Intoxication†	166 (4.0)	43 (6.9)
Complications†	44 (1.1)	12 (1.9)
Abdominal	495 (10.9)	91 (13.4)
Other	568 (12.5)	94 (13.9)

†Not available in Denmark

## Discussion

The main result of this study was that metabolic bariatric surgery at age > 60 years was associated with a decreased mortality compared to non-operative treatment for obesity.

Main strengths of the study include the population-based design and the large sample size of patients with metabolic bariatric surgery at older age, which was achieved through nationwide inclusion from three countries. We used data from national registries which have been largely validated for the purposes of the study, reducing the risk of misclassification and selection. Due to the large sample size of older operated patients, we could use both matching and extensive adjustments for obesity-related diseases and frailty variables to reduce confounding. We could also analyse mortality in women and men separately and patients in different age strata with retained precision. We were aware of the potential risk of confounding through selection of healthier individuals to have metabolic bariatric surgery at older age and therefore took meticulous care to reduce its impact. Following matching, we did not find any clear signs that patients that underwent surgery were healthier at baseline apart from a slightly lower prevalence of cardiovascular disease. As we observed this, we performed a post-hoc analysis which demonstrated that metabolic bariatric surgery was associated with decreased mortality after exclusion of patients with cardiovascular disease at baseline, suggesting robustness of the main results.

While known confounding was likely well controlled after adjustments, we lacked data on preoperative body mass index, smoking, and socioeconomic status, which was not available from the data sources. While these variables may be associated with both exposure and outcomes, they were indirectly adjusted for by the inclusion of closely associated diseases (e.g., diabetes and chronic obstructive pulmonary disease) in the statistical model. The direction of bias from any residual confounding by body mass index or smoking is not immediately clear. Presumably, patients who underwent operation might have more severe obesity compared to non-operated, while active smoking may be a reason to not operate. We attempted to account for any selection of healthier individuals by adjusting for three separate variables associated with frailty, i.e., previous pneumonia, hospitalizations, and thrombosis. These variables were similarly distributed among operated and non-operated patients at baseline, suggesting no major differences in frailty. Nevertheless, it cannot be ruled out that frailty was not completely captured by these three variables and that there remained residual confounding. Finally, we did not have data on the precise treatment for obesity in the non-operative group. With these limitations in mind, the study should be internally valid and the nationwide inclusion from three countries suggests excellent generalizability to other countries with similar demographics and healthcare structure.

Few studies have examined long-term mortality rates specifically in older patients undergoing metabolic bariatric surgery. A US cohort study performed in a high-volume metabolic bariatric surgery centre examined mortality outcomes by different age strata in patients with gastric bypass. Of these, 1210 patients were operated at age 55 or older and followed for mean 5.9 years, where mortality was 50% decreased (HR 0.50, 95% CI 0.31–0.79) compared to age-matched controls with obesity [22]. The association found in that study was slightly more pronounced than in the current study, which may have been due to overall younger age in the American cohort or by the inclusion of gastric bypass procedures only. Metabolic bariatric surgery performed in high-volume centres is also associated with improved long-term survival, which would also explain more moderate associations observed in the current study, which included all metabolic bariatric surgeries in the participating countries (including from low volume centres) [23]. From this study, it appeared as if the protective association between metabolic bariatric surgery and mortality becomes weaker with older age as the benefit of surgery disappeared at age > 70 years. It should be noted that the study could not entirely rule out a small benefit for surgery in patients > 70 years because of limited precision. Also, patients with metabolic bariatric surgery had a considerably better prognosis with lower mortality in the first 15 years of follow-up. After this period, the adjusted mortality rates were similar among operated and non-operated patients, meaning that the effect size of the protective association between metabolic bariatric surgery and mortality waned with longer follow-up time.

There are reports of reduced benefits of metabolic bariatric surgery in older patients, including less weight loss and lower rates of resolution of obesity-related diseases such as diabetes and hypertension [24]. The consequences of the reduced impact of metabolic bariatric surgery on weight and obesity-related diseases were also largely verified in a recent population-based cohort study where the incidence of cancer (HR 0.81, 95% CI 0.64–1.03) and cardiovascular disease (HR 0.86, 95% CI 0.74–1.01) were similar in patients with metabolic bariatric surgery at age 60 years or older compared to matched controls [25]. While the results of that study were non-significant, point estimates indicated a slightly decreased risk of both outcomes, which are major causes of death for older patients with obesity. This was also verified in this study, were both operated and non-operated patients largely died due to diseases related to old age, e.g., cardiovascular disease and cancer, and to a less degree related to intoxication, which is a more common cause of death in younger individuals with metabolic bariatric surgery [26]. Protective associations were particularly pronounced in patients with diabetes and in those operated in more recent calendar periods, which may be explained by a higher proportion of patients with gastric bypass in these strata. This notion was also verified in the subgroup analysis limited to patients with gastric bypass, where the association with decreased mortality was profound. It should be noted that perioperative mortality in this study was clearly higher than what has been reported in younger individuals (typically < 0.1%), which highlights the need for preoperative risk assessment and careful selection of older individuals for surgery [27].

In conclusion, this population-based cohort study of older patients with severe obesity found metabolic bariatric surgery to be associated with improved mortality and prolonged life expectancy. These benefits did not appear to extent to patients of age 70 years or older but were more profound in patients with diabetes. The findings from this study suggests a role for metabolic bariatric surgery as a procedure to prolong life expectancy in older patients with severe obesity.

## Supplementary Information

Below is the link to the electronic supplementary material.

Supplementary file1 (DOCX 29 KB)

## Data Availability

Data may be obtained from a third party and are not publicly available. Protocols and statistical analysis plans will be shared upon reasonable request to the corresponding author.
