# Supplementary material for: Mortality and Causes of Death After Metabolic Bariatric Surgery in Older Patients
Source: Obes Surg. 2026 Jan 12;36(3):941–7. doi: 10.1007/s11695-026-08487-7 (PMC13038464; doi:10.1007/s11695-026-08487-7)
Supplement: Supplementary file 1 — Supplementary file1 (DOCX 29 KB) [file 11695_2026_8487_MOESM1_ESM.docx]

**Supplementary document for manuscript “Mortality and Causes of Death After Metabolic Bariatric Surgery in Older Patients”.**

**Index**

**Supplementary Table 1.** *Procedure Codes For Metabolic Bariatric Surgery.*

**Supplementary Table 2.** *ICD-Codes For Obesity.*

**Supplementary Table 3.** *ICD-Codes For Comorbidities.*

**Supplementary Table 4.** *Causes Of Death In Older Patients With Metabolic Bariatric Surgery And Non-Operative Treatment For Obesity That Occurred Within 90 Days From Study Entry.*

**Supplementary Table 5.** *Mortality In Older Patients Operated With Gastric Bypass And Nonoperative Treatment For Obesity.*

| **Supplementary Table 1. Procedure Codes For Metabolic Bariatric Surgery.** | | | |
| --- | --- | --- | --- |
| **Operation** | **NOMESCO* (1997-)** | **Classification of Operations (Swe, 1963-1996)** | **Finnish Hospital League classification (1986-1996)** |
| Vertical-Banded Gastroplasty |  | 4751 |  |
| Gastric Bypass | JDF10-11, JDF50-51 | 4752 |  |
| Gastric Banding | JDF20-21 | 4753 |  |
| Sleeve Gastrectomy | JDF96 (after 2000), JDF97 (after 2000), JDF40-41, JDF00-01 (after 2000) | - | - |
| Duodenal Shunt with Biliopancreatic Diversion | JFD03-04 | 4750 |  |
| Other | JDF00-01 | 4759 | 6548, 6559 |
| *From 1996 in Denmark. Patients entered the study in 1996 in Denmark because metabolic bariatric surgery could not be identified before the introduction of NOMESCO. | | | |

| **Supplementary Table 2. ICD-Codes For Obesity.** | | | | |
| --- | --- | --- | --- | --- |
|  | **ICD-7** | **ICD-8** | **ICD-9** | **ICD-10** |
| Any Country | 287 | 277 | 278A | E66 |
| Sweden |  |  | 278A, 278B |  |
| Finland |  |  | 2780, 2781 |  |

| **Supplementary Table 3. ICD-Codes For Obesity- and Frailty-Related Diseases.** | | |
| --- | --- | --- |
| **Comorbidity** | **ICD-9** | **ICD-10** |
| Diabetes | 250 | E11-E14 |
| Hypertension | 401-405 | I10-I15 |
| Peripheral Vascular Disease | 440–447, V43E, 785E | I70–I73, I770–I771, K551, K558– K559, R02, Z958–Z959 |
| Chronic Obstructive Pulmonary Disease and Other Smoking-Related Lung Disorders | 466, 490-492, 494, 496 | J40-J41, J43-J44, J47 |
| Renal Disease | 403– 404, 580–588, V42A, V45B | I12–I13, N01, N03, N05, N07–N08, N171–N172, N18, N19, N25, Z49, Z940, Z992 |
| Cardiovascular Disease | 410-414, 430–438, 362D, 342-344 | I20-I25, G45–G46, I60–I69 |
| Deep Vein Thrombosis or Pulmonary Embolism | 415B, 416W, 451B | I802, I26 |
| Pneumonia | 480-486 | J12-18 |

**Supplementary Table 4. Causes Of Death In Older Patients With Metabolic Bariatric Surgery And Non-Operative Treatment For Obesity That Occurred Within 90 Days From Study Entry.**

|  | **Deaths, No. (%)** |  |
| --- | --- | --- |
| **Cause of death** | **Non-Operative Treatment** | **Metabolic Bariatric Surgery** |
| Total | 74 | 24 |
| Cardiovascular | 48 (64.9) | 13 (54.4) |
| Cancer | 8 (10.8) | 4 (16.7) |
| Infections | 6 (8.1) | <4 (<5.0) |
| Intoxication† | <5 (<10.0) | <4 (<5.0) |
| Complications† | <4 (<5.0) | 0 (0.0) |
| Abdominal | 8 (10.8) | 10 (41.7) |
| Other | 16 (21.6) | <4 (<10.0) |

† Not available in Denmark

**Supplementary Table 5. Mortality In Older Patients Operated With Gastric Bypass And Non-Operative Treatment For Obesity**

|  | **Non-Operative Treatment** | |  | **Gastric Bypass** | | | |
| --- | --- | --- | --- | --- | --- | --- | --- |
|  |  |  |  |  |  | **Hazard Ratio (95% CI)** | |
|  | **Person-years** | **Deaths, No.** |  | **Person-years** | **Deaths, No.** | **Unadjusted** | **Adjusted** |
| Entire follow-up | 111806 | 3158 |  | 23862 | 455 | 0.66 (0.61-0.73) | 0.64 (0.58-0.71) |
| Follow-up (years) |  |  |  |  |  |  |  |
| >0-1 | 13793 | 240 |  | 2779 | 20 | 0.41 (0.26-0.65) | 0.42 (0.27-0.66) |
| >1-5 | 55127 | 1216 |  | 11525 | 137 | 0.54 (0.45-0.64) | 0.53 (0.44-0.64) |
| >5-10 | 31588 | 1124 |  | 6995 | 179 | 0.72 (0.61-0.84) | 0.68 (0.58-0.79) |
| >10 | 11298 | 578 |  | 2564 | 119 | 0.91 (0.75-1.10) | 0.84 (0.69-1.03) |

Model 1: Crude

Model 2: Adjusted for diabetes, hypertension, peripheral vascular disease, chronic obstructive pulmonary disease, renal disease, cardiovascular diseases, deep vein thrombosis or pulmonary embolism, pneumonia, number of previous hospital admissions.

The proportionality of the hazard was checked calculating the Schoenfeld residuals for the crude model for the entire follow-up time analysis. The assumption of proportionality was not valid.
